# Reversal of Myopic Correction for Patients Intolerant to LASIK

**DOI:** 10.1155/2021/7113676

**Published:** 2021-12-15

**Authors:** Amr A. Gab-Alla

**Affiliations:** Ophthalmology Department, Suez Canal University, Faculty of Medicine, Ismailia, Egypt

## Abstract

**Purpose:**

To evaluate the outcome of the reversal of myopia correction in patients intolerant to LASIK.

**Methods:**

This study is a retrospective and case series of patients who decided to reverse their previous myopic LASIK correction between July 2012 and July 2020. It was conducted at a private refractive surgery centre, Ismailia, Egypt. The patients were followed up after reversal treatment for one year. Primary LASIK and reversal treatment were performed by a 500 kHz Amaris excimer laser platform. The main outcomes included refractive predictability, stability, efficacy, and safety and any reported complications.

**Results:**

This study included 48 eyes of 24 patients (6 male and 18 female patients). The average duration between the primary LASIK surgery and reversal treatment was 3.20 ± 0.30 months (range 3 to 4 months). Reversal treatment was bilateral in all patients. The mean age of the patients was 38 ± 1.9 years (range 37 to 45 yrs). After reversal, the mean postreversal cycloplegic refraction spherical equivalent was −1.82 ± 0.34 D (range −1.50 to −3.00 D). The mean ablation depth was 34.10 ± 7.36 *μ*m (range 20 to 46 *μ*m), and the mean of the central corneal thickness 12 months after reversal treatment was 510.2 ± 14.4 *μ*m (range 515 to 487 *μ*m). The mean keratometric reading was 42.6 ± 1.6 (range 42.5 to 44.8). The mean of CDVA was 0.2 ± 0.03 log MAR (range −0.10 to 0.4 log MAR). The mean optical zone of reversal treatment was 6.1 ± 0.3 mm (range 5.9 to 6.2 mm). UDVA was 0.4 log MAR in 87.5% of the patients, 0.5 log MAR in 8.3% of the patients, and 0.6 log MAR in 4.2% of the patients. CDVA remained unchanged in 83.3% of patients; 2.1% of the patients gained one line of CDVA (Snellen); 8.3% of the patients lost one line of CDVA; 6.3% of the patients lost two lines of CDVA. No cases of corneal ectasia were recorded. The only postoperative complications were flap microfolds in 3 eyes (6.25%).

**Conclusion:**

In conclusion, this study demonstrates that reversal of myopic LASIK treatment is a safe, stable, and effective option for intolerant patients.

## 1. Introduction

Laser-assisted in situ keratomileusis (LASIK) is the most common refractive procedure to correct different refractive errors, including hyperopia, myopia, and astigmatism [1]. It has been reported to improve patient-reported visual outcomes significantly [1]. The result is the patient's freedom from contact lenses and glasses [2]. It has been shown in several studies to enhance not only visual function^1^ but also patient quality of life (QOL) [3–6].

Unless the patient's expectations of the procedure were unreasonable, patient satisfaction should be high after an uneventful refractive surgery with a good objective result. The relationship between patient expectation and education, patient satisfaction, and surgical outcome, on the other hand, is complicated [7]. Frequently, refractive results do not correlate well with satisfaction. Failure to conduct a thorough subjective evaluation can also prevent the visualization of particular dissatisfaction areas [8–10]. In their analysis, Kahle and coauthors [8] discovered that 84% of patients were pleased with their myopic correction after surgery, whereas 16% were indifferent or dissatisfied. El-Maghraby et al. [9] found that 90.5% were pleased, and 9.5% were unhappy overall. There have been reports of at least temporary losses of low-contrast visual acuity and contrast sensitivity [11–14].

The root of any disappointment must be identified in order to achieve optimal postoperative satisfaction levels. This involves holding a detailed conversation with prospective patients on all facets of their everyday lives and hobbies in order to assess their motivations for having surgery and their post-LASIK aspirations [14].

The rating of intolerance to LASIK was unaffected by preoperative refraction [4]. Women, on the other hand, were more likely than men to report intolerance to optical aids [4]. However, clinical experience indicates that it may be related to women reporting a higher rate of contact lens-induced dry eye [15]. However, patient intolerance seems to be age-related, with younger patients showing slightly higher levels of satisfaction than older patients [10]. Different treatment options for ophthalmologists are currently available. With rising patient expectations, secure and successful outcomes are critical, and they can correct or reverse the results of a previous LASIK procedure if the patient is unhappy [16].

One of the major concerns of LASIK is the reversibility of the procedure. However, further studies are needed to ensure the visual outcomes of this reversal profile. For these reasons, we believe that further investigation is needed to improve our knowledge about the real advantages and disadvantages of the reversibility of LASIK treatment of unsatisfied myopic patients. So, this study aimed at evaluating the outcome of reversal of myopic correction in patients intolerant to LASIK.

## 2. Methods

This study is a retrospective and case series conducted at a private refractive eye centre, Ismailia, Egypt, between July 2012 and July 2019. Unsatisfied patients who had previously undergone bilateral myopic LASIK procedures met the following inclusion criteria: reduced near vision and poor near activities, IOP less than 21 mm Hg, CCT > 500 m at the periphery of the cornea, measured residual stromal bed after treatment > 60% of total corneal thickness, and a normal corneal topography pattern (Sirius, CSO, Florence, Italy). There is no history of diabetes or autoimmune disorders. A trial of a monovision contact lens was tried before bilateral reversal of myopic LASIK for all patients. Patients who did not receive adequate follow-up were removed from the study.

Standard LASIK procedures were used to perform the primary LASIK. The Moria M2 microkeratome was used to make the corneal flap (Moria, Antony, France). A 500 kHz Amaris E excimer laser was used for laser ablation (Schwind eye-tech-solutions, Kleinostheim, Germany). The research protocol was approved by the Suez Canal University Faculty of Medicine's institutional ethical review board (Approval No. 4655), and the study followed the Declaration of Helsinki's tenets. Before reversing the previous myopic LASIK surgery, all patients signed a written informed consent form.

### 2.1. Reversal of Myopic LASIK

Relifting the corneal flap was used in reversal procedures. After the eye was anaesthetized with two drops of 0.4% benoxinate hydrochloride at 5-minute intervals, the flap edge was identified at the slit lamp and marked with a pen marker. After that, the patient was taken to the laser bed. Using a flat spatula, the flap edge was completely removed from the bed. Sweep movement was performed from the flap pedicle to the periphery to separate the flap from the corneal bed. After the excimer laser treatment, the flap was replaced. In all patients, the Schwind Amaris E 500 kHz LASIK platform was used to ablate the corneal stroma at a 6.0-mm optical zone. All surgeries targeted mild myopia for the near based on the patients' age and native refraction with correction range from +1.5 to +2.5 D.

Postoperatively, the patients were examined on the 1st day, 1st week, 1st, 3rd, 6th months, and one year after the surgery. The patients were assessed by complete ocular examinations. Refraction (cycloplegic and manifest in spherical equivalent), keratometry, corneal thickness, corrected distance visual acuity (CDVA), uncorrected distance visual acuity (UDVA), and corneal topography were the main outcome measures. Reversal surgery was performed after the third-month follow-up visit following the primary LASIK procedure (when the patient was dissatisfaction with the visual result). One physician operated on the patients, examined them, and followed up with them (AAG).

### 2.2. Statistical Analysis

The Statistical Package for the Social Sciences (SPSS) version 25 was used to manipulate and analyze all of the data (IBM Corporation, NY, USA). The study groups' parameters were shown as frequencies and percentages, as well as mean values and standard deviations. The Kolmogorov Smirnov test was used to determine if the data were normal. The differences in means between pre- and post-intervention measures were compared using the paired *t*-test. Repeated measures ANOVA was used to compare the differences in mean measurements over time. Graph Pad Prism (version 5.00 for Windows, Graph Pad Software, La Jolla California USA) and Microsoft Excel (version 2016) were used to construct the graphs. A *p* value less than 0.05 was considered statistically significant.

## 3. Results

This study included 48 eyes of 24 patients (6 male and 18 female patients) who decided to reverse their previous myopic LASIK correction. Before LASIK, the mean cycloplegic refraction spherical equivalent was −2.50 ± 0.90 D (range −1.75 to −3.50 D). The average duration between the primary LASIK surgery and reversal treatment was 3.2 ± 0.3 months (range 3 to 4 months). Reversal treatment was bilateral in all patients. The mean age of the patients was 38 ± 1.9 years (range 37 to 45 yrs).

Before reversal, the mean cycloplegic refraction spherical equivalent was +0.25 ± 0.80 D (range +0.50 to −0.50 D). The mean of the central corneal thickness was 510.3 ± 8.3 *μ*m (range 520 to 490 *μ*m). The mean keratometric reading was 41.0 ± 1.4 (range 39.5 to 41.6). The mean of UDVA was 0.15 ± 0.07 log MAR (range 0.00 to 0.5 log MAR). The mean optical zone of LASIK treatment was 6.2 ± 0.2 mm (range 5.9 to 6.4 mm).

After reversal, the mean postreversal cycloplegic refraction spherical equivalent (SE) was −2.40 ± 0.26 D (range −1.50 to −3.00 D). The mean ablation depth was 34.10 ± 7.36 *μ*m (range 20 to 46 *μ*m), and the mean of the central corneal thickness 12 months after reversal treatment was 510.2 ± 14.4 *μ*m (range 515 to 487 *μ*m). The mean keratometric reading was 42.6 ± 1.6 (range 42.5 to 44.8). The mean of CDVA was 0.2 ± 0.03 log MAR (range −0.10 to 0.4 log MAR). The mean optical zone of reversal treatment was 6.1 ± 0.3 mm (range 5.9 to 6.2 mm) (Table 1).

### 3.1. Refractive Predictability

**Figure 1:**
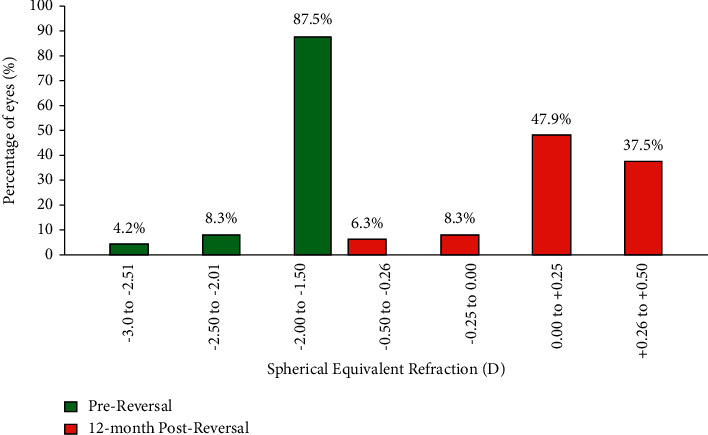
Distribution of mean refractive spherical equivalent (MRSE) (predictability) at 12 months after reversal treatment.

**Figure 2:**
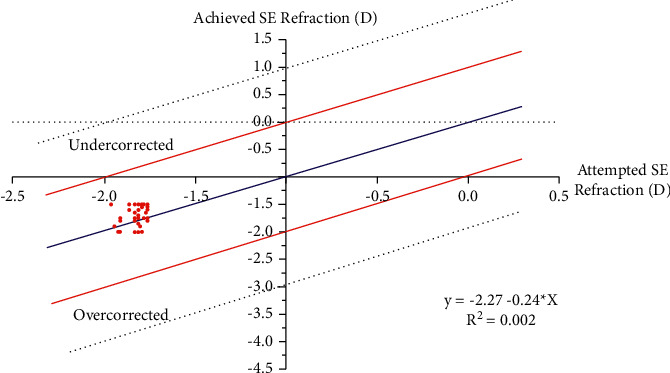
Scatterplot of the attempted spherical equivalent (SE) correction versus the achieved (SE) correction 12 month after reversal of LASIK.

At the 12th month after reversal treatment, the mean of refractive spherical equivalent (MRSE) was −1.82 ± 0.34 D (range −1.50 to −3.00 D). Before reversal, 6.3% of the patients were within −0.50 to −0.25 D, 8.3% of the patients were within −0.25 to 0.00 D of target refraction, 47.9% of the patients were within 0.00 to +0.25 D, and 37.5% of the patients were within +0.25 to +0.50 D. After reversal treatment, 87.5% were within −1.50 to −2.00 D, 8.3% were within −2.01 to −2.50 D and 4.2% were within −2.51 to −3.00 D. The distribution of MRSE before and after reversal treatment can be found in Figure 1.  Figure 2 shows the scatterplot of the attempted SE correction versus the achieved SE correction 12 months after reversal treatment.

### 3.2. Stability

**Figure 3:**
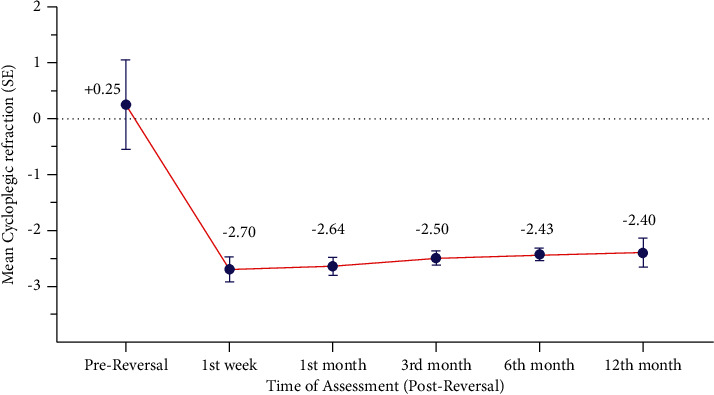
Refractive stability after reversal treatment (12 months).

Postreversal treatment data were reported at the 1st week, 1st month, 3rd month, 6th month, and 12th month. The mean of the spherical equivalent refraction showed statistical significant changes (*P* < 0.0001) from post-LASIK +0.25 ± 0.80 D (range +0.50 to −0.50 D) to −2.40 ± 0.26 D (range −1.75 to −3.50 D) at 1^st^ week, −2.64 ± 0.17 D (range −1.50 to −3.50 D) at 1 month, −2.50 ± 0.13 D (range −1.50 to −3.25 D) at 3 months, −2.43 ± 0.11 D (range −1.50 to −3.00 D) at the 6th month, and −2.40 ± 0.26 D (range −1.50 to −3.00 D) at the 12th month (Table 2, Figure 3).

### 3.3. Visual Acuity and Efficacy

**Figure 4:**
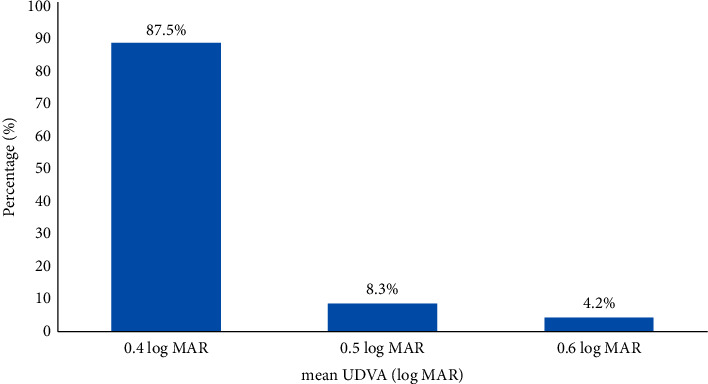
Mean of UDVA (log MAR) at 12th month after reversal treatment.

After reversal treatment, mean UDVA (log MAR) was significantly changed at the 1st week, 1st month, 3rd month, 6th month, and 12th month to 0.62 ± 0.10, 0.52 ± 0.7, 0.43 ± 0.11, 0.42 ± 0.06, and 0.40 ± 0.55, respectively (Table 3). At the 12th month after reversal treatment, UDVA was 0.4 log MAR in 87.5% of the patients, 0.5 log MAR in 8.3% of the patients, and 0.6 log MAR in 4.2% of the patients (Figure 4).

### 3.4. Safety

**Figure 5:**
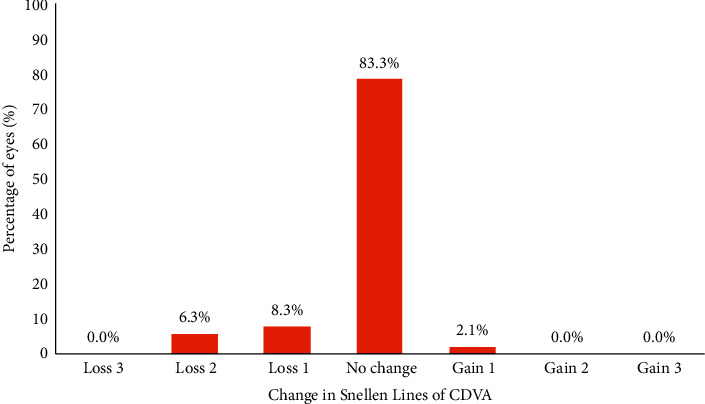
Change in lines of CDVA at 12th month postreversal treatment (safety).

The safety of the treatment was assessed at the 12th month of follow-up, CDVA remained unchanged in 83.3% of patients; 2.1% of the patients gained one line of CDVA (Snellen); 8.3% of the patients lost one line of CDVA (Snellen); 6.3% of the patients lost two lines of CDVA (Snellen) (Figure 5).

### 3.5. Complications

No cases of corneal ectasia were recorded. The only postoperative complications were flap microfolds in 3 eyes (6.25%). No additional treatment was required in these cases, which improved at the end of the follow-up time.

## 4. Discussion

In ophthalmic practice and research, it is becoming increasingly clear that taking into account a patient's assessment of their functioning and symptoms is critical when determining the need for and outcome of care. In recent years, the demand for myopia refractive surgery has risen significantly. This is due to objective performance in terms of better-unaided vision and reduced refractive error, but it depends on patient satisfaction after surgery [16]. Patient satisfaction has been shown to be a valuable indicator of outcome in the evaluation of the quality of treatment, contact patterns, utilization of services offered, and those delivering such services in a variety of clinical fields [16]. As a result, it is important for those involved in the care of refractive surgery patients to identify the reasons for having surgery as precisely as possible, as this can have a significant impact on postoperative patient satisfaction [16].

The litigious nature of our culture necessitates thorough and accurate informed consent from prospective patients versus the clinician's view of success before surgery. However, as the types of patients that come in for surgery become more varied in terms of their refractive errors, so might their reasons for seeking the surgery and, therefore, to some extent, their expectations of the refractive surgery [17]. Realistic expectations have been shown to correlate well with postoperative patient satisfaction [18]. Thus, if appropriate informed consent and treatment are to be offered, a better understanding of the rationale for receiving this type of care is needed.

Patients who are highly motivated and take risks are thought to have a significant influence on their decision to have LASIK [19]. These patients are often armed with a wealth of information, not just about their future treatment, but also about their surgeon and the hospital where they will be treated [19]. As a result, it is fair that physicians become equally astute about their patients' needs to attempt to satisfy them.

Additionally, an increase in optical aberrations has been observed after LASIK [20]. The majority of the contrast sensitivity loss after LASIK is due to an increase in optical aberrations, which is attributed to a loss of low-contrast visual acuity [20]. After LASIK, some patients subjectively experience night-vision symptoms such as glare, halos, and starbursts, in addition to changes in objective tests of visual function such as contrast sensitivity [21]. The natural aspheric shape of the cornea is thought to reduce some of the eye's optical aberrations [21]. Corneas become more oblate after myopic LASIK, according to research [20]. Increases in asphericity (more oblate) have also been shown in calculated models to increase the Seidel spherical aberration of the eye [21]. As a result, a cornea that is already flatter or less prolate than usual before LASIK surgery can be more vulnerable to increased optical aberrations (and therefore glare, halos, and starbursts) after the operation [21].

Overall, subjects in this study were unsatisfied with their vision after LASIK. Age is linked to lower satisfaction after LASIK surgery, as has been previously stated [22, 23]. This result may not be a problem specific to LASIK. Increased age is linked to higher scores on the Refractive Status and Vision Profile (RSVP) questionnaire for symptoms, visual issues, and corrective lens problems, according to Vitale et al. [24]. Furthermore, around the age of presbyopia, we do not fully satisfy the optical needs of our presbyopic patients or that these patients are having trouble accommodating due to the current refractive status. It could also be the result of the decline in optical performance that already occurs with age [25].

In this study, the time for doing the reversal treatment was 3.20 ± 0.30 months (range 3 to 4 months) because the patients were unsatisfied with their vision immediately after myopic LASIK correction. Previous studies [26–29] showed the stability of refraction after LASIK at the 3rd month postoperative. In studies performed by Pe´rez-Santonja et al. [30], they enhanced the patients at the 3rd month after the primary LASIK, Brahma et al. [31] enhanced the patients at the 4th week after the primary LASIK. Also, Lyle and Jin [32] retreated their patient from the 3rd month after the primary LASIK. On the other hand, Andreas et al. [33] concluded in their study on hyperopic astigmatic LASIK or photorefractive keratectomy that keratometric changes are followed by refractive changes after corneal laser refractive surgery, and they occur up to 6 months after LASIK and for at least 6 months after photorefractive keratectomy. As a result, caution should be used when retreatment is planned during the first year after surgery, because hyperopic refractive regression can result in suboptimal visual outcomes.

Due to the correlation with night-vision problems, a flatter corneal curvature was found to be a factor associated with reduced patient satisfaction after LASIK. Preoperative minimum corneal curvature values were substantially lower in subjects who had starbursts [34]. However, there was no clear “cut point” for corneal curvature at which patients seemed to be unhappy [34].

Before LASIK surgery, patients should be properly counselled about the risk of experiencing new visual effects due to the operation. Although visual symptoms were common after LASIK surgery, only a few people said they had a significant effect [34]. The limited number of reports of the more troublesome symptoms precluded evaluating correlations with other factors. Postoperative satisfaction was found to correlate with UCVA after LASIK in agreement with previous studies [35–37] that demonstrated reduced postoperative UCVA due to residual refractive error as a common reason for dissatisfaction.

Lazon De la Jara et al. [38] reported that visual acuity (VA) measures at all contrast levels were moderately associated with the frequency of disturbing visual and ocular symptoms. We can deduce from these findings that VA as tested only described a portion of the patient's visual problems during everyday activities. This result emphasizes the importance of using quality of life (QOL) and self-assessment tests for refractive error in refractive surgery preoperative and postoperative exams to gather useful and additional knowledge about visual status. The cognitive dissonance theory refers to a psychological process that creates a change in attitudes and behaviour to maintain a cognitive consistency towards their beliefs [39]. This effect will be more pronounced when patients pay a fee for the operation, and the surgery is reversible.

Satisfaction post-LASIK is mainly correlated with improved visual function, psychological characteristics, patients' preoperative expectations, and UCVA achieved. The ophthalmological community would be wise to abandon the use of only two objectively determined biological variables, UCVA and residual refractive error, as benchmarks for evaluating refractive surgery outcomes. A precise evaluation of the subjective visual quality and patient satisfaction following refractive surgery is needed as myopic patients who need refractive surgery may have different psychological profiles and standards than those who need other ophthalmic procedures [19].

This study aimed at evaluating the outcomes of surgical reversal of myopia in patients intolerant to LASIK. At present, scientific reports on this topic are rare, with only case studies [16, 40, 41]. This is the first case series report of reversal myopia treatment that we are aware of. This research is significant because it allows other surgeons to do future LASIK reversals if needed. Limitations of the study are given as follows: first, this was a retrospective study, and in this way, all related restrictions must be thought of; and second, the follow-up time is relatively short. With this short period of follow-up, one cannot talk about ectasia and its percentage. Third, this study used topography data that only have an average asphericity value for the entire cornea. This averaging may have resulted in the loss of more extreme values, resulting in a smaller gap in corneal asphericity between those who experience night-vision symptoms and those who do not.

In conclusion, this study demonstrates that reversal of myopic 400 LASIK treatment is a safe, stable, and effective option for intolerant 401 patients.

## Figures and Tables

**Table 1:** Baseline characteristics of the study groups.

Characteristics	Prereversal LASIK group	LASIK reversal group
Refraction SE (D)	+0.25 ± 0.80 (+0.50 to –0.50)	–2.4 ± 0.26 (–1.50 to –3.00)
Mean ± SD		
Range		

CCT (*μ*m)	510.3 ± 8.3 (520 to 490)	510.2 ± 14.4 (515 to 487)
Mean ± SD		
Range		

K Readings (D)	41.0 ± 1.4 (39.5 to 41.6)	42.6 ± 1.6 (42.5 to 44.8)
Mean ± SD		
Range		

Optical zone (mm)	6.2 ± 0.2 (5.9 to 6.4)	6.1 ± 0.3 (5.9 to 6.2)
Mean ± SD		
Range		

Abbreviations: SE: spherical equivalent; CCT: central corneal thickness; K reading: keratometry reading; D: dioptre; *μ*m: micrometre.

**Table 2:** Postreversal cycloplegic refraction outcomes spherical equivalent (SE) in dioptres.

Time of follow-up	Cycloplegic refraction (SE)
	Mean ± SD
	Range
Before reversal	+0.25 ± 0.80 D (+0.50 to −0.50 D)
First week after reversal	−2.70 ± 0.22 D (−1.75 to −3.5 D)
First month after reversal	−2.64 ± 0.17 D (−1.50 to −3.5 D)
Third month after reversal	−2.50 ± 0.13 D (−1.50 to −3.25 D)
Sixth month after reversal	−2.43 ± 0.11 D (−1.50 to −3.00 D)
12th month after reversal	−2.40 ± 0.26 D (−1.50 to −3.00 D)
*P* value	<0.0001^*∗*^

*Note.* SE: spherical equivalent. ^*∗*^Statistically significant.

**Table 3:** 12-month postreversal treatment log MAR of uncorrected distance visual acuity (UDVA).

Time of follow-up	Uncorrected distance visual acuity (log MAR)
	Mean ± SD
	Range
Before reversal	0.06 ± 0.04 (0.2 to −0.1)
First week after reversal	0.62 ± 0.10 (0.6 to 1.00)
First month after reversal	0.52 ± 0.7 (0.6 to 0.90)
Third month after reversal	0.43 ± 0.11 (0.50 to 0.90)
Sixth month after reversal	0.42 ± 0.06 (0.50 to 0.90)
12th month after reversal	0.40 ± 0.55 (0.50 to 0.90)
*P* value	<0.0001^*∗*^

*Note.* UDVA: uncorrected distance visual acuity. ^*∗*^statistically significant.

## Data Availability

The datasets used and/or analyzed during the current study are available upon request to the author.
